# A Real-World Evaluation of Clinical Prognostic Scores in Advanced Melanoma Treated with Immune Checkpoint Inhibitors

**DOI:** 10.3390/jcm14186452

**Published:** 2025-09-12

**Authors:** Gul Sema Yildiran Keskin, Nuri Karadurmus

**Affiliations:** Department of Medical Oncology, Gulhane Training and Research Hospital, Etlik, Ankara 06010, Turkey; drnkaradurmus@yahoo.com

**Keywords:** metastatic melanoma, immune checkpoint inhibitors, prognostic scores, RMH score, GRIm score, MDA-ICI score

## Abstract

**Background**: Despite improved survival outcomes with immune checkpoint inhibitors (ICIs), the treatment response of patients with metastatic melanoma remains highly variable. There is a growing need for reliable, accessible prognostic tools that incorporate clinical and inflammatory markers in order to stratify patients better and guide therapeutic decisions. **Methods**: We conducted a retrospective cohort study involving 73 patients with metastatic cutaneous melanoma treated with ICIs at a single tertiary center between 2017 and 2024. Baseline clinical and laboratory parameters were collected to calculate the Royal Marsden Hospital (RMH), Gustave Roussy Immune (GRIm) and MD Anderson Immune Checkpoint Inhibitor (MDA-ICI) scores. Overall survival (OS) and progression-free survival (PFS) were analyzed via Kaplan–Meier estimates and Cox proportional hazards models. Prognostic performance was assessed using Harrell’s concordance index (C-index) and receiver operating characteristic (ROC) analysis. **Results**: The median follow-up was 35.9 months, and the median OS was 22.1 months. All three scores were significantly associated with OS in univariate analysis. In multivariate models, only the RMH (HR: 5.45, *p* < 0.001) and MDA-ICI (HR: 4.24, *p* = 0.015) scores remained independent predictors of OS. Harrell’s C-index indicated strong discriminative ability for both RMH (0.742) and MDA-ICI (0.730) scores, whereas the GRIm score demonstrated lower predictive accuracy (0.615). Similarly, ROC curve analysis showed higher AUC values for RMH (0.732) and MDA-ICI (0.739) compared with GRIm (0.595). **Conclusions**: In this real-world cohort of metastatic melanoma patients treated with ICI, the RMH and MDA-ICI scores demonstrated favorable prognostic performance and outperformed the GRIm score in predicting overall survival. These findings support the clinical utility of RMH and MDA-ICI as practical, accessible tools for prognostic risk stratification in melanoma, though external validation in larger, multicenter cohorts is required.

## Simple Summary

Immune checkpoint inhibitors (ICIs) have significantly improved survival outcomes in patients with advanced melanoma. However, predicting treatment response remains challenging. In this real-world study, we evaluated three prognostic models: the Royal Marsden Hospital (RMH) score, the Gustave Roussy Immune (GRIm) score, and the MD Anderson Immune Checkpoint Inhibitor (MDA-ICI) score. These models incorporate clinical and laboratory data that are routinely measured in oncology practice. Our results indicate that the RMH and MDA-ICI scores provide the most accurate prognostic stratification, whereas the GRIm score showed limited utility in this setting.

## 1. Introduction

Malignant melanoma is an aggressive cancer that primarily affects the skin (cutaneous melanoma), and less commonly, the mucosal or uveal tissues. While the global incidence of cutaneous melanoma has been steadily increasing, mortality rates have declined in recent decades due to significant therapeutic advances [1,2]. Metastatic melanoma treatment currently relies on immune checkpoint inhibitors (ICIs) and targeted therapies, with BRAF and MEK inhibitors used in patients with BRAF V600E mutations [3,4]. Five-year overall survival rates are approximately 40% with anti-PD-1 monotherapy and up to 50% with combination immunotherapy using anti-CTLA-4 plus anti-PD-1 antibodies [5,6,7,8,9]. Despite these therapeutic advances, a substantial proportion of patients still fail to respond to immunotherapy, and response rates remain difficult to predict. These findings underscore the need for prognostic and predictive biomarkers to guide individualized treatment strategies.

Tumor-induced immunosuppression within the tumor microenvironment constitutes a pivotal mechanism in cancer progression. This process is often driven by systemic and chronic inflammation, which is mediated by various circulating immune cells [10]. Numerous inflammatory and immune-related markers have been investigated for their prognostic significance. The neutrophil-to-lymphocyte ratio (NLR) has demonstrated prognostic significance across multiple solid tumors [11]. In melanoma, elevated NLR and systemic immune-inflammation index (SII = NLR × platelet count) have been identified as independent predictors of reduced survival in patients treated with ipilimumab or nivolumab [12,13,14,15,16].

Serum lactate dehydrogenase (LDH) represents one of the most widely recognized and validated prognostic biomarkers in metastatic melanoma. Beyond its classical interpretation as a surrogate of tumor burden, LDH is now understood to an play active role in cancer metabolism, immune evasion, and oncogenic signaling, making it a biologically and clinically relevant marker in immuno-oncology [17,18].

In addition to inflammatory markers, established clinical factors including the number and sites of metastases, along with Eastern Cooperative Oncology Group (ECOG) performance status, serve as prognostic indicators in patients with melanoma [16,19,20,21,22]. Beyond single biomarkers, several composite prognostic scores integrating clinical and laboratory variables have been developed to improve risk stratification. The Royal Marsden Hospital (RMH) score and the MD Anderson Cancer Center (MDACC) prognostic score were the first to combine clinical and laboratory variables and have been widely used for patient selection in clinical trials [23,24]. More recently, immune-specific scoring systems have been developed to optimize patient selection in the immunotherapy era. The Gustave Roussy Immune (GRIm) score and the MD Anderson Immune Checkpoint Inhibitor (MDA-ICI) score were specifically designed for ICI-treated populations [25,26]. Several retrospective studies have demonstrated their prognostic value in real-world ICI-treated cohorts beyond early-phase clinical trials [27,28,29].

Various biomarker-driven models have also been proposed to assess survival outcomes in metastatic melanoma patients treated with ICIs. These include models based on PD-L1 expression, tumor mutational burden (TMB), and gene expression profiles [30,31,32,33]. However, many of these approaches require advanced molecular testing or are not easily applicable in routine clinical settings due to cost and technical complexity.

In contrast, the RMH, GRIm, and MDA-ICI scores are based on routinely available clinical and laboratory parameters, making them feasible and cost-effective for daily oncology practice. These models offer several advantages. They are reproducible and easily calculated. Importantly, they have demonstrated consistent associations with survival outcomes in ICI-treated cohorts, which makes them ideal candidates for real-world validation [34].

In this study, we aimed to evaluate and compare the prognostic performance of these three scoring systems in a real-world cohort of metastatic melanoma patients treated with ICIs. We also assessed the discriminatory ability of these systems for survival outcomes and their applicability in routine clinical practice.

## 2. Methods

### 2.1. Patient Selection

We retrospectively analyzed a continuous cohort including all eligible metastatic melanoma patients who received first-line ICI therapy at the Gulhane Training and Research Hospital between 2017 and 2024. The relatively limited sample size reflects the real-world constraints of immunotherapy access in Turkey due to reimbursement policies during the study period, which restricted access to combination immunotherapy as first-line treatment for some patients. Ethical approval was obtained from the institutional ethics committee (Approval number: 2025-276; date of approval: 6 May 2025).

The inclusion criteria were as follows: histologically confirmed malignant melanoma, administration of first-line ICI therapy, ECOG performance status of 0–3, and absence of active autoimmune disease. The exclusion criteria were insufficient follow-up duration and missing treatment response data.

### 2.2. Laboratory and Score Calculations

Prior to the initiation of immunotherapy, baseline laboratory parameters were collected, including the absolute counts of neutrophils, monocytes, platelets, and lymphocytes, as well as LDH, CRP, and albumin levels.

The RMH score was based on the number of metastatic sites and the serum albumin and LDH values. The GRIm score was based on the NLR, LDH, and albumin levels. The MDA-ICI score was calculated with age, ECOG PS, liver metastases, LDH, and the absolute platelet, neutrophil, and lymphocyte counts. The components and risk categorization of the prognostic scoring systems are shown in Table 1.

### 2.3. Statistical Analysis

Descriptive statistics were used to summarize the patient characteristics and baseline variables. Continuous variables were reported as medians with interquartile ranges (IQRs; 25th–75th percentiles), while categorical variables were presented as counts and frequencies.

PFS was defined as the time from the initiation of immunotherapy to either disease progression, death, or the last follow-up. OS was defined as the time from the initiation of immunotherapy to death or the last follow-up, whichever occurred first. Survival outcomes were estimated using the Kaplan–Meier method, and differences between subgroups were evaluated with the log–rank test.

Variance inflation factor (VIF) values were calculated to assess multicollinearity among the prognostic scores. A VIF value below 5 was considered acceptable, with values under 3 indicating low collinearity. In our analysis, all three scores, RMH, GRIm, and MDA-ICI, demonstrated VIF values < 3, suggesting no significant multicollinearity.

Univariate and multivariate Cox proportional hazards models were used to calculate hazard ratios (HRs) with 95% confidence intervals (CIs). For the multivariate analysis, only the prognostic scores (RMH, GRIm, and MDA-ICI) were included to avoid multicollinearity, as individual clinical variables (ECOG performance status, number of metastatic organs, liver metastasis) are integral components of these composite scores, and their inclusion would introduce collinearity.

The discriminatory ability of the prognostic scores was assessed using both Harrell’s concordance index (C-index) and ROC curve analysis. Harrell’s C-index was calculated based on Cox proportional hazards models to evaluate the model’s concordance between the predicted and observed survival times. ROC curve analysis was performed for overall survival status at last follow-up (exitus: yes/no) to determine the area under the curve (AUC), 95% confidence intervals, and *p* values.

All statistical analyses were performed using IBM SPSS Statistics for Windows, version 25.0 (IBM Corp., Armonk, NY, USA). Harrell’s C-index was calculated via the lifelines package in Python (version 3.13.3, Python Software Foundation, Wilmington, DE, USA).

A two-tailed *p*-value < 0.05 was considered statistically significant for univariate analyses. Bonferroni correction was applied for the three pairwise score comparisons in both Cox regression analyses and ROC AUC comparisons, resulting in an adjusted significance threshold of *p* < 0.0167.

## 3. Results

A total of 73 patients with cutaneous malignant melanoma who received first-line immune checkpoint inhibitors for metastatic disease were included in this study. The median age was 64 years (IQR: 52.5–74), 53.4% were male, 47.9% were older than 65 years, and 43.8% had de novo metastatic disease. The majority of patients (72.6%) had an ECOG PS of 0–1. A BRAF V600E mutation was detected in 21.9% of patients, and 13.7% received BRAF plus MEK inhibitors as adjuvant therapy. None of the patients had received adjuvant immunotherapy before developing metastatic disease. Nivolumab monotherapy was administered to 80.8% of the patients, while 19.2% received nivolumab plus ipilimumab. Baseline patient and treatment characteristics are summarized in Table 2.

**Figure 1:**
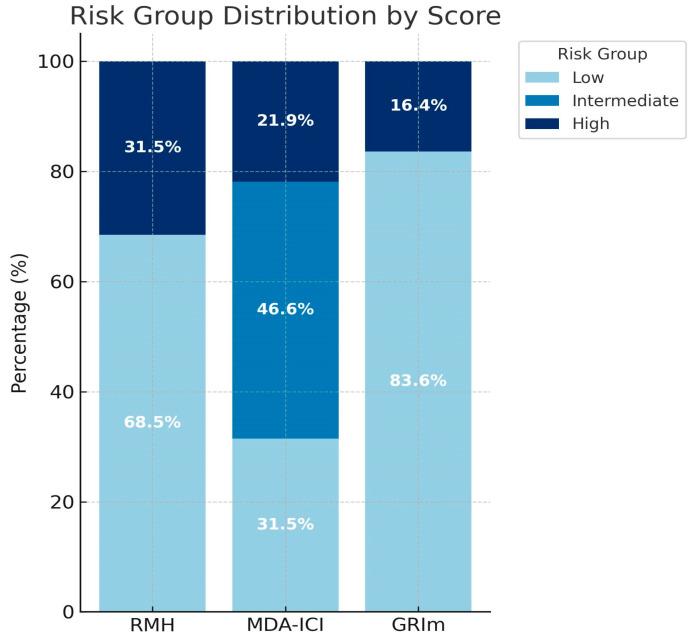
Distribution of patients according to prognostic risk groups.

Risk group distribution varied across the three scoring systems. According to RMH, 68.8% were classified as low risk and 31.5% as high risk. Based on MDA-ICI, 31.5% were low risk, 46.6% intermediate risk, and 21.9% high risk. For GRIm, 83.6% were low risk and 16.4% were high risk. Risk group distributions are shown in Figure 1.

### 3.1. Survival Outcomes

**Figure 2:**
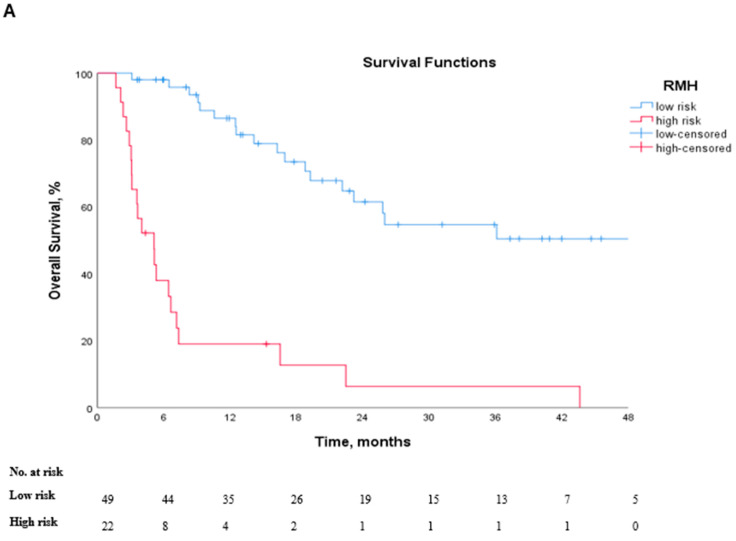

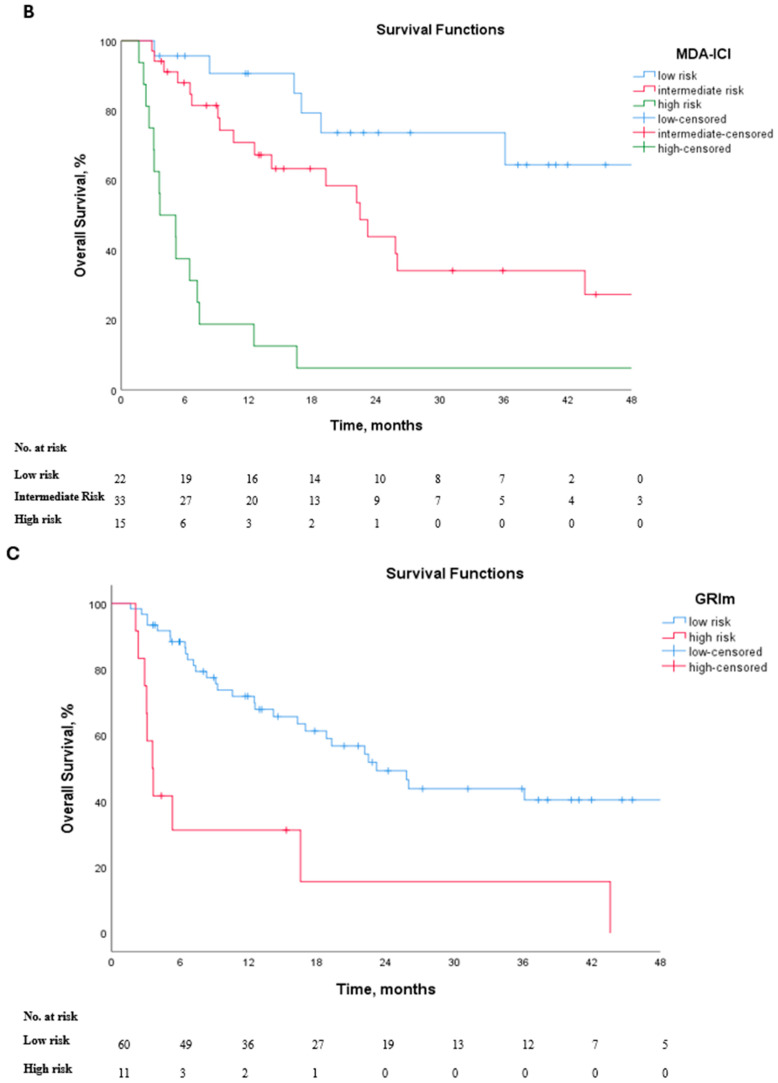
Kaplan–Meier curves for overall survival based on prognostic scoring systems. (**A**) RMH score, (**B**) MDA-ICI score, and (**C**) GRIm score. In all comparisons, higher risk scores were associated with significantly worse survival outcomes (*p* < 0.001 for all comparisons).

The median was 35.9 months (95% CI 20.4–51.3 months). Median PFS was 10.8 months (95% CI: 4.9–16.7), and median OS was 22.1 months (95% CI: 15.1–29). Overall survival differed significantly across risk groups, defined by each scoring system (*p* < 0.001 for all comparisons). For the RMH score, median OS was 72.9 months in the low-risk group versus 5.1 months in the high-risk group. Similarly, the GRIm score demonstrated median OS values of 23.1 months and 3.5 months in the low- and high-risk categories, respectively. In the case of the MDA-ICI score, the median OS decreased progressively across the risk groups: 72.9 months for low-risk patients, 19.6 months for intermediate-risk patients, and 3.6 months for high-risk patients. The Kaplan–Meier survival curves illustrating OS according to each prognostic scores are shown in Figure 2A–C.

In univariate Cox analyses, the ECOG PS, number of metastatic sites, liver metastasis, and all three prognostic scores were significantly associated with OS. Univariate analyses results are provided in Supplementary Table S1. In multivariable Cox regression, a high RMH score was independently associated with significantly shorter OS (HR: 5.45; 95% CI: 2.13–13.96; *p* < 0.001). Similarly, patients classified as high-risk by the MDA-ICI score (score 5–7) had worse OS compared with those in the low-risk group (HR: 4.25; 95% CI: 1.33–13.55; *p* = 0.015). In contrast, the GRIm score did not retain statistical significance in the multivariate model (*p* = 0.532). These findings indicate that the RMH and MDA-ICI scores provide independent prognostic information in ICI-treated patients with metastatic melanoma. Multivariate Cox regression results for OS are presented in Table 3.

### 3.2. Prognostic Performance of Clinical Risk Scores

**Figure 3:**
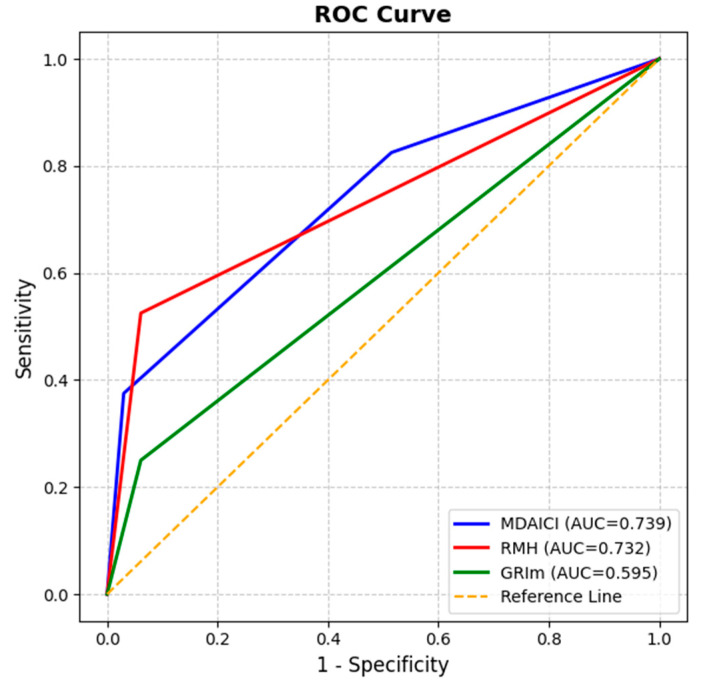
Receiver operating characteristic (ROC) curves of prognostic scores for overall survival prediction. The RMH and MDA-ICI scores showed relatively higher AUC values (0.732 and 0.739, respectively) compared with the GRIm score, which demonstrated a lower AUC (0.595). The area under the curve (AUC) reflects the discriminative capacity of each score in distinguishing survival outcomes.

The discriminative ability was evaluated with Harrell’s C-index and ROC curve analysis (Table 3; ROC curves in Figure 3). Among the three scores, RMH showed the strongest prognostic performance (C-index: 0.742; AUC 0.732 [95% CI: 0.616–0.848]; *p* = 0.001). MDA-ICI also demonstrated good discriminative ability (C-index: 0.730; AUC 0.739 [95% CI: 0.626–0.852]; *p* < 0.001). In contrast, the GRIm score showed limited discrimination (C-index: 0.615) and failed to reach statistical significance based on ROC analysis (AUC = 0.595, *p* = 0.166). This suggests limited utility for guiding decision-making in patients with metastatic melanoma treated with ICIs. To control type-1 error, Bonferroni correction was applied across the three score comparisons (adjusted α = 0.0167); RMH and MDA-ICI remained significant after correction.

## 4. Discussion

In this retrospective cohort study conducted in a real-world setting, we evaluated and compared the prognostic performance of three clinical scoring systems, RMH, GRIm, and MDA-ICI, in patients with metastatic malignant melanoma treated with immune checkpoint inhibitors. Our findings demonstrated that RMH and MDA-ICI scores outperformed GRIm score in prognostic discrimination and remained independently associated with overall survival in multivariate analysis.

Although the GRIm score was not significant in our multivariate model, its prognostic value has been reported in several real-world ICI-treated populations [35]. Ma et al. observed the superior discriminatory performance of GRIm compared with RMH, MDA, and MDA-ICI scores in patients with metastatic gastric and esophageal cancer [34]. Similarly, Shangguan et al. showed that the GRIm could serve as a useful prognostic tool in small-cell lung cancer patients treated with PD-1/PD-L1 inhibitors [36]. However, in melanoma, studies have demonstrated inconsistent results. GRIm’s reliance solely on laboratory parameters (NLR, LDH, and albumin) without including clinical factors such as performance status or metastatic burden may explain its limited prognostic performance in melanoma. Indeed, functional status has consistently been shown to be a critical determinant of survival in metastatic melanoma [37].

Conversely, RMH and MDA-ICI integrate both laboratory and clinical variables, allowing a more comprehensive evaluation of tumor burden, systemic inflammation, and patient condition. The RMH score combines LDH, serum albumin, and the number of metastatic sites, while the MDA-ICI incorporates additional parameters such as ECOG performance status, age, liver metastases, and peripheral blood counts. This multidimensional design likely contributes to their stronger prognostic capacities, especially in melanoma, where host–tumor interaction and inflammatory response are tightly linked. Nevertheless, the prognostic performance of RMH has varied across tumor types and treatment settings. For instance, Al Darazi et al. externally validated several scores in ICI phase-I trials and found that GRIm and LIPI (Lung Immune Prognostic Index, composed of LDH and derived NLR) outperformed RMH [38]. Likewise, in a prospective longitudinal study, García-Corbacho et al. reported LIPI to be superior to RMH and GRIm for predicting early progression, OS, and PFS across metastatic solid tumors, whereas RMH was associated only with durable clinical benefit [39]. In contrast, in ICI-resistant advanced melanoma, Acar et al. confirmed that a higher RMH score independently predicted shorter OS in both anti-PD-1 monotherapy and nivolumab–ipilimumab cohorts and was associated with PFS in the combination cohort [27]. Taken together, while RMH retains strong disease-specific utility in melanoma, its broader applicability in pan-cancer ICI contexts may be limited, and complementary biomarker-driven or dynamic models warrant further evaluation.

Importantly, the significant variability in OS observed in our cohort further emphasizes the need for reliable, accessible prognostic tools to guide clinical decision-making. These scores can help identify patients who could benefit from early escalation to combination immunotherapy or integration of targeted therapy, while also supporting timely referral to supportive and palliative care in those with poor prognoses.

Grad et al. demonstrated that high-risk metastatic melanoma patients, as identified by clinical and laboratory-based models, were significantly more likely to receive ICI therapy at the end of life [40]. This highlights the importance of timely risk stratification to prevent futile late-line interventions and to ensure that treatment decisions are aligned with patient prognosis and care goals.

Diem et al. proposed a prognostic score based on LDH, ECOG performance status, and number of involved organs in advanced melanoma patients receiving ipilimumab, which demonstrated clear separation in survival outcomes across risk groups [41]. Although this model is not identical to RMH, it shares core components. Our study also found that RMH showed strong prognostic value (HR: 5.45, *p* = 0.001), supporting the reproducibility and clinical relevance of these parameters in real-world clinical settings.

This study has several limitations. First, it was a retrospective, single-center analysis with a relatively small sample size, which may limit the generalizability of the results. Because of the retrospective design and fixed sample size, no a priori power calculation was performed, and as with all retrospective analyses, the potential impact of unmeasured confounding factors cannot be excluded. External validation in larger multicenter cohorts is warranted. Second, reimbursement restrictions in our country during the study period led to an imbalanced treatment distribution, with nearly 80% of patients receiving anti-PD-1 monotherapy (nivolumab) and only 20% receiving nivolumab plus ipilimumab. This limits the applicability of our findings to combination ICI regimens, which are increasingly used in eligible high-risk patients. Third, we included patients with ECOG PS up to 3 to better reflect real-world practice. This approach enhances the external validity of our findings by representing a broader patient population than that of typical clinical trials. However, it may also limit direct comparability with trial populations that usually restrict enrollment to ECOG PS 0–1 and could influence outcome interpretation. Finally, molecular biomarkers such as PD-L1 expression, tumor mutational burden, and gene expression profiles were not assessed, limiting comparison with biomarker-driven prognostic models.

In conclusion, the RMH and MDA-ICI scores that integrate both clinical and laboratory parameters appear to provide more reliable prognostic stratification than GRIm in patients with metastatic melanoma treated with ICIs. Their applications in clinical practice may help identify patients who could benefit from early escalation to combination immunotherapy or de-escalation or discontinuation of immunotherapy and timely integration of supportive and palliative care. Prospective validation in larger multicenter cohorts is warranted to confirm their clinical utility.

## Figures and Tables

**Table 1:** Components and risk categorization of the prognostic scores.

Prognostic Scores	Components	Risk
Royal Marsden Hospital score (RMH)	>2 metastatic sites = 1 point Serum albumin < 35 g/L = 1 point LDH > ULN = 1 point	0–1: Low risk 2–3: High risk
Gustave Roussy Immune Score (GRIm)	Serum albumin < 35 g/L = 1 point LDH > ULN = 1 point NLR > 6 = 1 point	0–1: Low risk 2–3: High risk
MD Anderson Immune Checkpoint Inhibitor Score (MDA-ICI)	Age > 52 years = 1 point ECOG > 1 = 1 point LDH > 0.75 × ULN = 1 point Platelets > 300 (10^3^/mL) = 1 point ANC > 4.9(10^3^/mL) = 1 point ALC < 1.8 (10^3^/mL) = 1 point Liver metastases = 1 point	0–2: Low risk 3–4: Intermediate risk 5–7: High risk

LDH: lactate dehydrogenase, ULN: upper limit of normal, ECOG: Eastern Cooperative Oncology Group, NLR: neutrophil-to-lymphocyte ratio, ANC: absolute neutrophil count, ALC: absolute lymphocyte count.

**Table 2:** Baseline patient and treatment characteristics.

Variables	N (%)
Age, median, IQR	64 (52.5–74)
<65	38 (52.1)
>65	35 (47.9)
Sex	
Female	34 (46.6)
Male	39 (53.4)
ECOG PS	
0–1	53 (72.6)
2–3	20 (27.4)
BRAF mutation	16 (21.9)
Denovo metastatic disease	32 (43.8)
Metastatic sites number	
1–2	53 (72.6)
>2	20 (27.4)
Metastatic site	
Lymph node	53 (72.6)
Lung	34 (46.6)
Liver	18 (24.7)
Brain	17 (23.3)
Bone	20 (27.4)
Type of immunotherapy	
Nivolumab	59 (80.8)
Ipilumumab + Nivolumab	14 (19.2)
Risk group distribution	
RMH score	
Low risk	50 (68.8)
High risk	23 (31.5)
MDA-ICI score	
Low risk	23 (31.5)
Intermediate risk	34 (46.6)
High risk	16 (21.9)
GRIm score	
Low risk	61 (83.6)
High risk	12 (16.4)

**Table 3:** Prognostic performance of clinical risk scores for overall survival (multivariate Cox regression and discrimination metrics).

Prognostic Score	HR (95% CI)	*p*-Value (Cox) *	Harrell’s C-Index	AUC (95% CI)	*p*-Value (ROC)*
RMH (high vs. low)	5.45 (2.13–13.96)	<0.001	0.742	0.732 (0.616–0.848)	0.001
MDA-ICI (int. vs. low) (high vs. low)	1.62 (0.60–4.40) 4.24 (1.33–13.55)	0.339 0.015	0.730	0.739 (0.626–0.852)	<0.001
GRIm (high vs. low)	0.75 (0.30–1.84)	0.532	0.615	0.595 (0.465–0.725)	0.166

Abbreviations: AUC: area under the curve; CI: confidence interval; ROC: receiver operating characteristic; RMH: Royal Marsden Hospital score; GRIm: Gustave Roussy Immune score; MDA-ICI: MD Anderson Immune Checkpoint Inhibitor score; Int: intermediate. * *p*-values were adjusted for multiple comparisons using the Bonferroni method. Three prognostic scores were compared (RMH, GRIm, and MDA-ICI), resulting in an adjusted significance threshold of *p* < 0.0167.

## Data Availability

The datasets generated and analyzed during the current study are not publicly available due to institutional privacy policies but are available from the corresponding author upon reasonable request.

## Supplementary Materials

The following supporting information can be downloaded at: https://www.mdpi.com/article/10.3390/jcm14186452/s1, Table S1: Univariate Cox analyses for overall survival.
