# Supplementary material for: A Real-World Evaluation of Clinical Prognostic Scores in Advanced Melanoma Treated with Immune Checkpoint Inhibitors
Source: J Clin Med. 2025 Sep 12;14(18):6452. doi: 10.3390/jcm14186452 (PMC12470320; doi:10.3390/jcm14186452)
Supplement: Supplementary file 1 [file jcm-14-06452-s001.zip › jcm-3788954-supplementary.pdf]

Supplementary Table S1. Univariate Cox analyses for overall survival.

| Variables | Univariate analysis | |
|---|---|---|
| | HR (95%CI) | P |
| Age | | |
| <65 vs ≥65 | 1.09 (0.58-2.05) | 0.788 |
| Sex | | |
| Female vs Male | 1.21 (0.64-2.28) | 0.547 |
| **ECOG PS** | | |
| **0-1 vs 2-3** | **6.53 (3.39-12.56)** | **<0.001** |
| BRAF mutation | | |
| Absence vs Presence | 1.08 (0.52-2.22) | 0.824 |
| Disease status | | |
| Denovo vs Recurrence | 0.81 (0.42-1.53) | 0.518 |
| Liver metastasis | | |
| Absence vs Presence | 4.11 (2.14-7.89) | <0.001 |
| **Number of Metastatic sites** | | |
| **1-2 vs >2** | **4.30 (2.26-8.17)** | **<0.001** |
| Type of ICI | | |
| Mono vs Combination ICI | 1.41 (0.61-3.23) | 0.411 |
| **RMH Score** | | |
| **Low vs High Risk** | **7.58 (3.92-14.68)** | **<0.001** |
| **GRIm Score** | | |
| **Low vs High Risk** | **3.97 (1.91-8.28)** | **<0.001** |
| **MDA-ICI Score** | | **<0.001** |
| **Low vs Intermediate Risk** | **2.37 (1.05-5.33)** | **0.037** |
| **Low vs High Risk** | **7.86 (3.28-18.81)** | **<0.001** |

Abbreviations: OS, overall survival; ECOG PS, Eastern Cooperative Oncology Group performance status; RMH, Royal Marsden Hospital score; GRIm, Gustave Roussy Immune score; MDA-ICI, MD Anderson Immune Checkpoint Inhibitor score.
